# Supplementary material for: Eugenol diffusion coefficient and its potential to control Sitophilus zeamais in rice
Source: Sci Rep. 2019 Aug 1;9:11161. doi: 10.1038/s41598-019-47562-1 (PMC6671970; doi:10.1038/s41598-019-47562-1)
Supplement: Supplementary file 1 — Supplementary information [file 41598_2019_47562_MOESM1_ESM.pdf]

Supplementary information

Eugenol diffusion coefficient and its potential to control *Sitophilus zeamais* in rice

Lucas Henrique Figueiredo Prates[a1], Lêda Rita D'Antonino Faroni[a2*], Fernanda Fernandes Heleno[b3], Maria Eliana Lopes Ribeiro de Queiroz[c4], Adalberto Hipólito de Sousa[a,d5], and Marcus Vinícius de Assis Silva[a6]

[a]Departamento de Engenharia Agrícola, Universidade Federal de Viçosa, Viçosa, 36570900, Minas Gerais, Brazil.

[b]Serviço Autônomo de Água e Esgoto, SAAE, Senador Firmino, 36540000, Minas Gerais, Brazil.

[c]Departamento de Química, Universidade Federal de Viçosa, Viçosa, 36570900, Minas Gerais, Brazil.

[d]Centro de Ciências Biológicas e da Natureza, Universidade Federal de Acre, Rio Branco, 69920900, Acre, Brazil.

*Corresponding author. Tel.: +55 (31) 3899-1874, Fax +55 (31) 3899-2735, lfaroni@ufv.br

ORCID, email:

[1]0000-0003-0739-5597, lucas.prates@ufv.br

[2]0000-0001-8648-5034, lfaroni@ufv.br

[3]0000-0002-0423-5662, fernandafhg@gmail.com

[4]0000-0003-4717-9223, meliana@ufv.br

[5]0000-0002-3089-2762, adalbertohipolito@hotmail.com

[6]0000-0002-5169-0203, marcus.assis@ufv.br

Correspondence: Lêda Rita D'Antonino Faroni, Departamento de Engenharia Agrícola, Universidade Federal de Viçosa, 36570-900, Viçosa, Minas Gerais, Brasil; Tel: + 55 31 3899-1874; e-mail: lfaroni@gmail.com

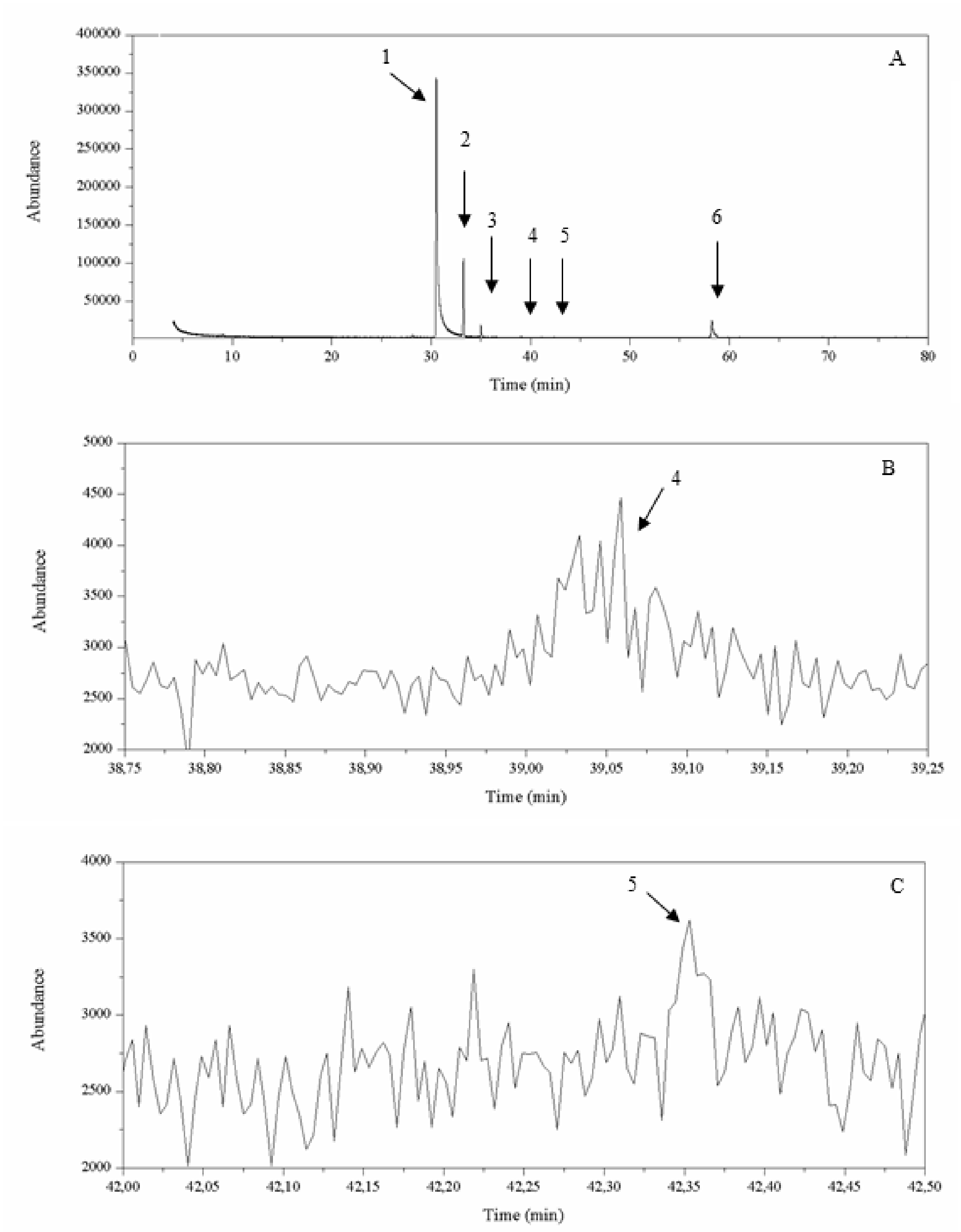

Figure S1 Chromatogram obtained from the injection of the essential oil of clove into gas chromatograph coupled to mass spectrometry (A), with zoom at the Retention time (Rt) related to the peaks number 4 (B) and number 5 (C). The numbers indicate the peaks of the following compounds and Rt (min), respectively: 1: eugenol, 30.55 min; 2: β-caryophyllene , 33.24 min; 3: α-caryophyllene, 35.01 min; 4: epizonarene, 39.06 min; 5: caryophyllene oxide, 42.35 min; 6: hexadecanol, 58.24 min.

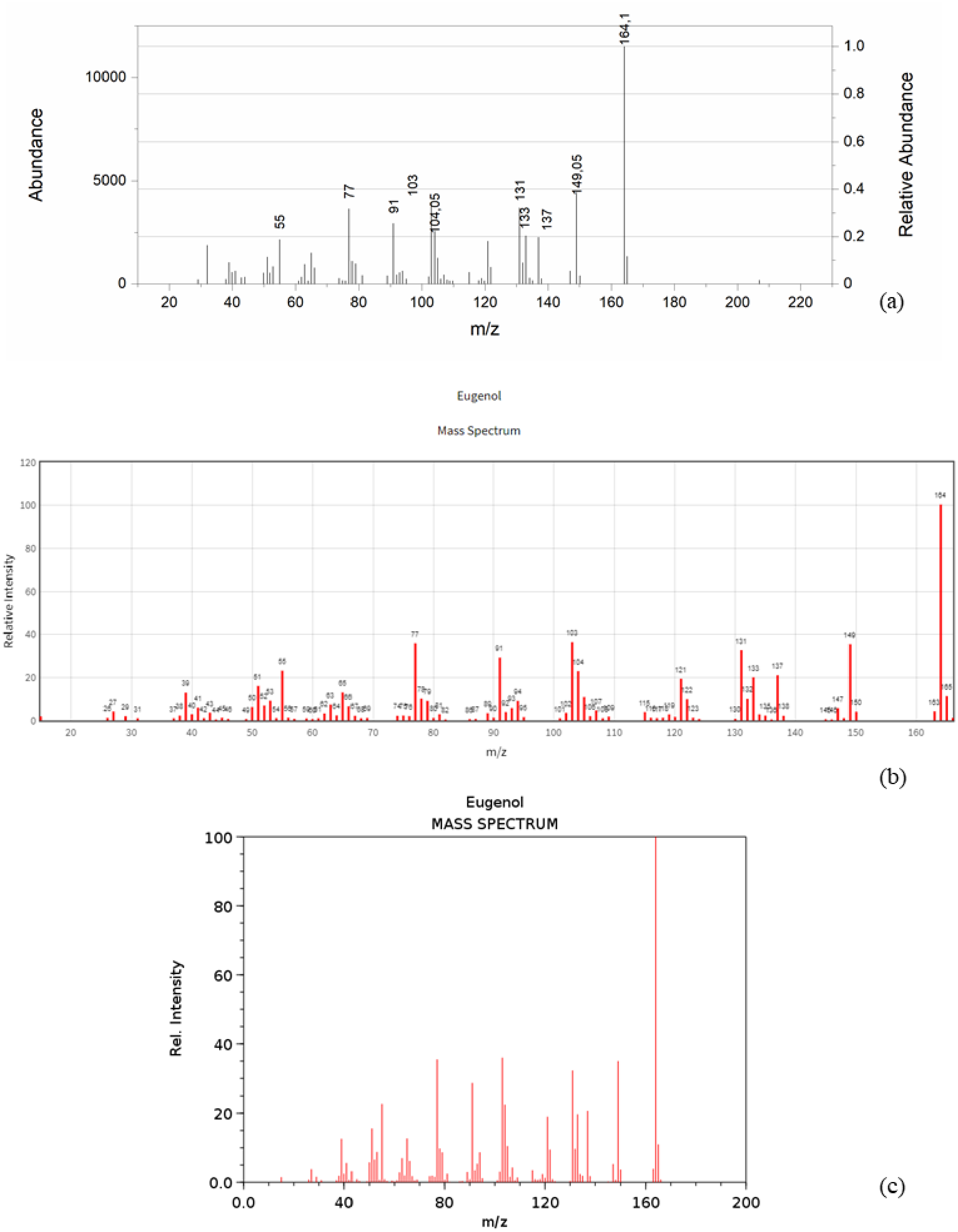

Figure S2 Mass to charge ratio (m/z) plot related to the chromatographic peak with retention time of 30.55 min (a), identified as eugenol, and the m/z plots for the identified compound according to the NIST library (b) and (c).

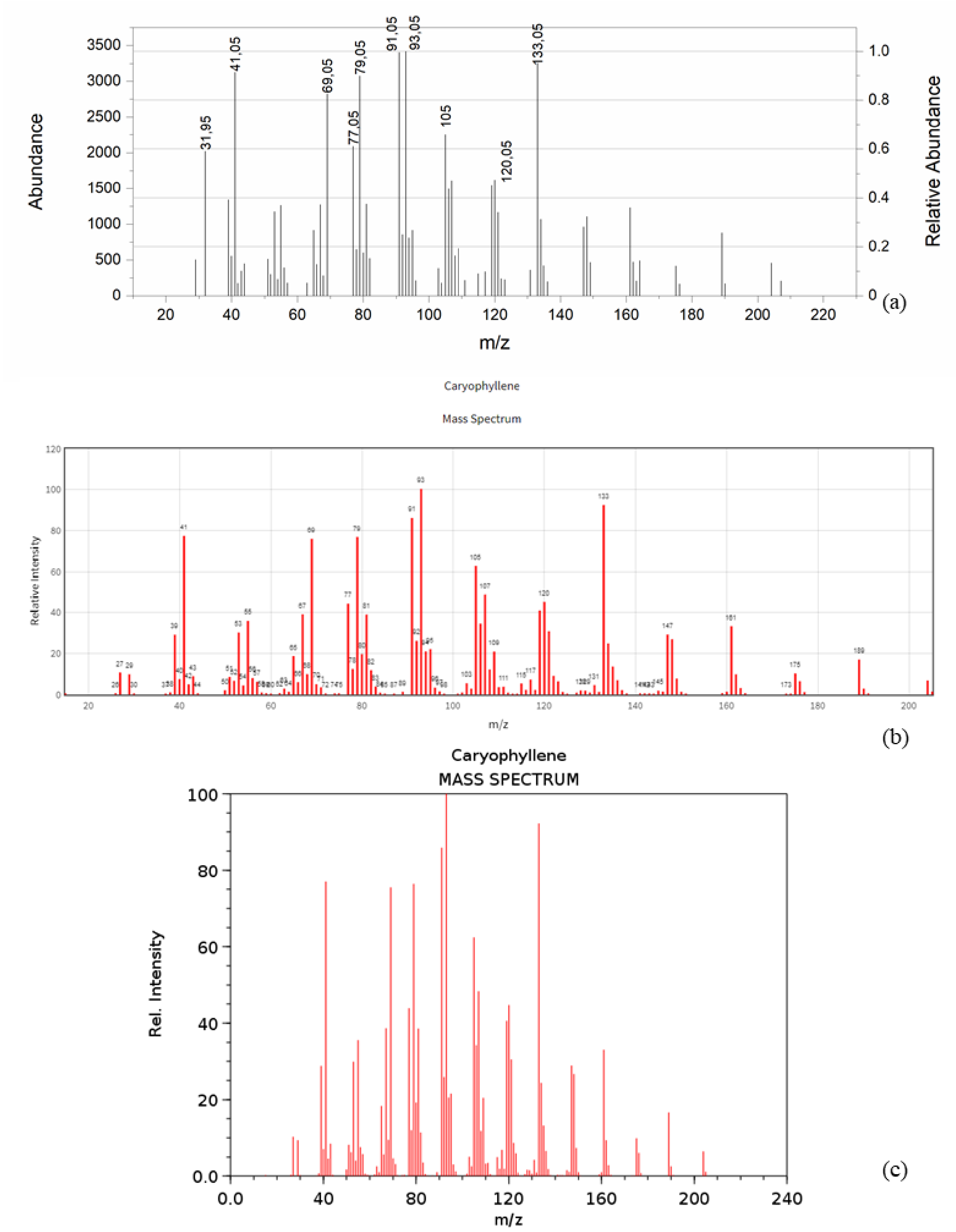

Figure S3 Mass to charge ratio (m/z) plot related to the chromatographic peak with retention time of 33.24 min (a), identified as β-caryophyllene (caryophyllene), and the m/z plots for the identified compound according to the NIST library (b) and (c).

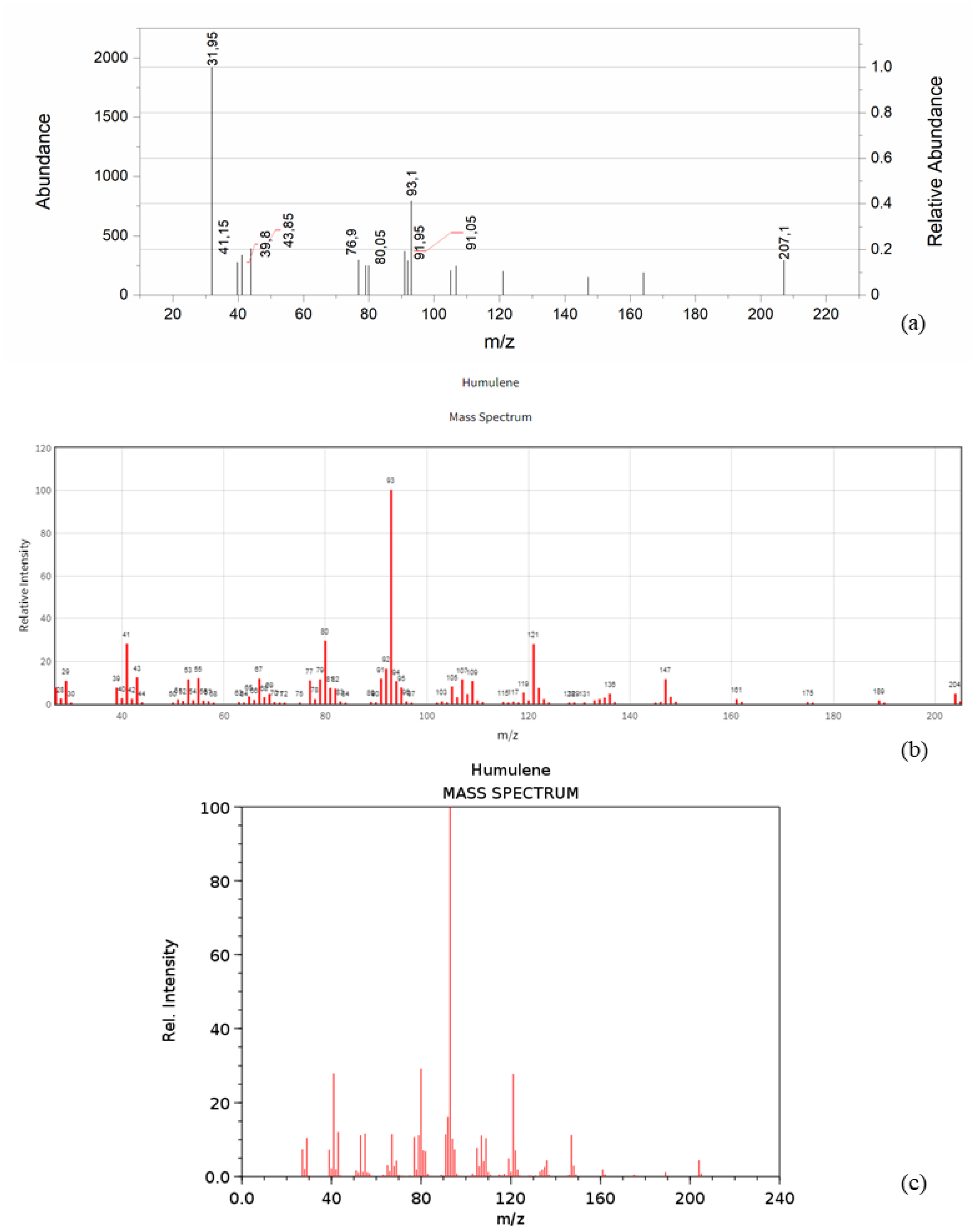

Figure S4 Mass to charge ratio (m/z) plot related to the chromatographic peak with retention time of 35.01 min (a), identified as α-caryophyllene (humulene), and the m/z plots for the identified compound according to the NIST library (b) and (c).

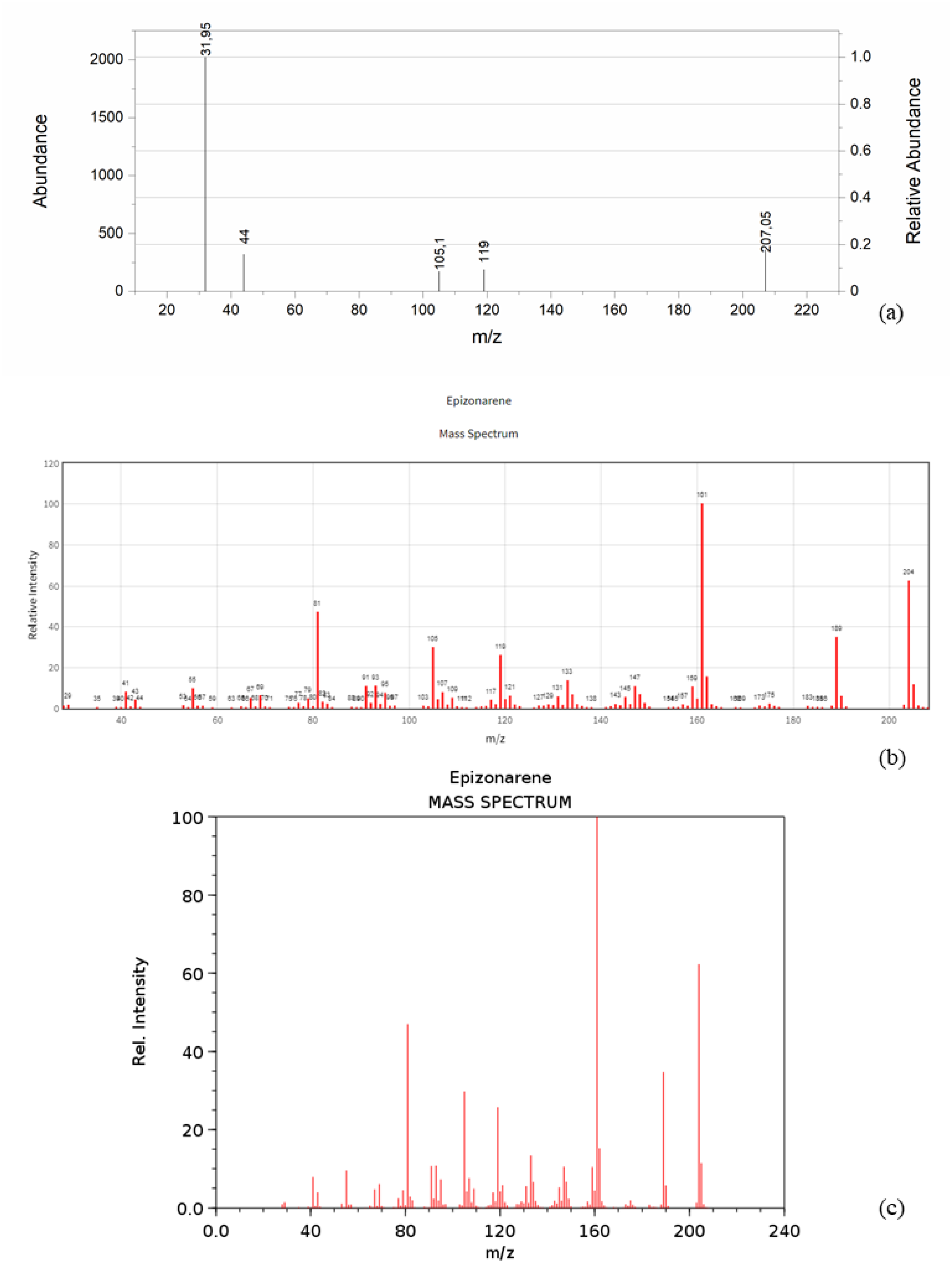

Figure S5 Mass to charge ratio (m/z) plot related to the chromatographic peak with retention time of 39.06 min (a), tentatively identified as epizonarene, and the m/z plots for the identified compound according to the NIST library (b) and (c).

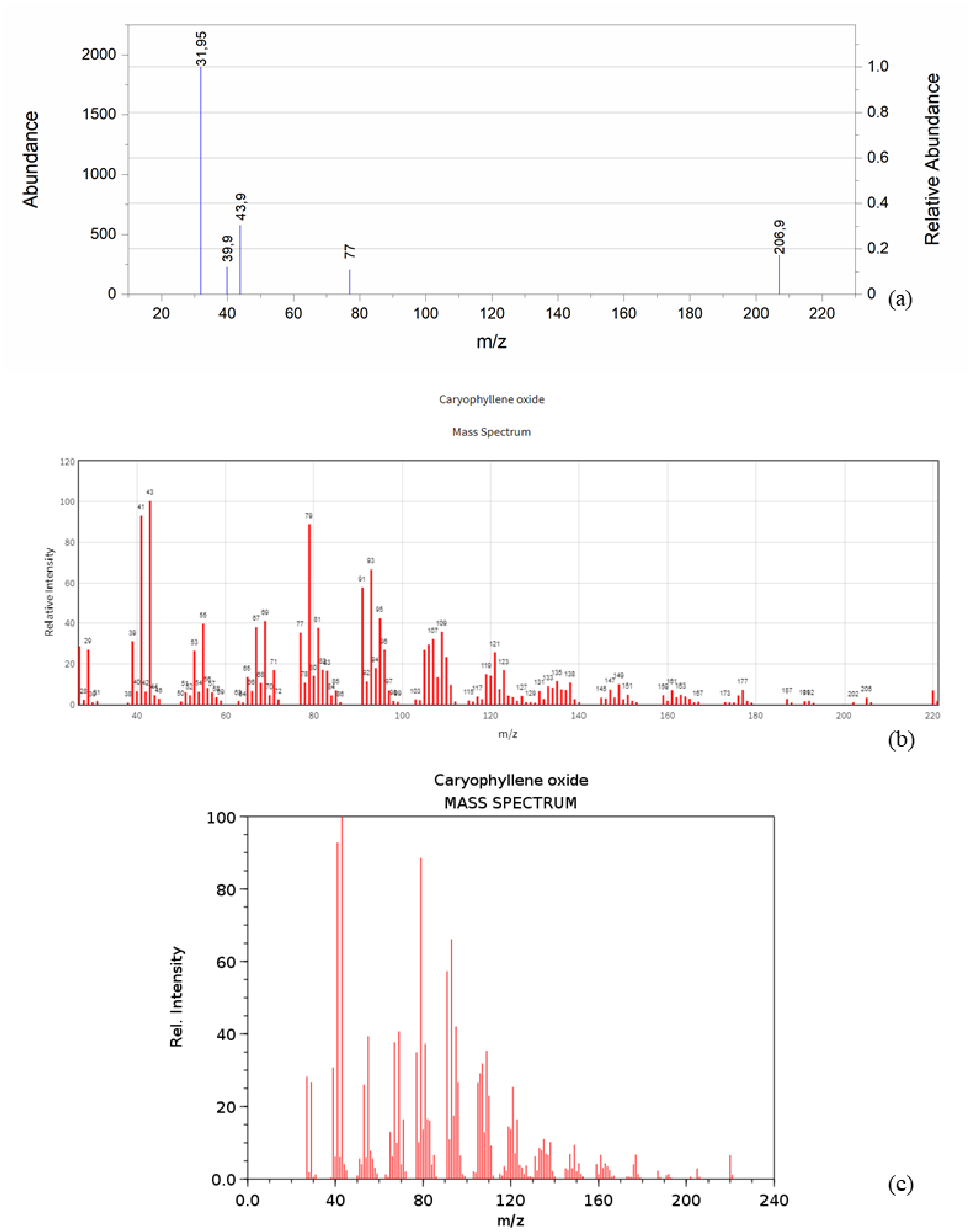

Figure S6 Mass to charge ratio (m/z) plot related to the chromatographic peak with retention time of 42.35 min (a), tentatively identified as caryophyllene oxide, and the m/z plots for the identified compound according to the NIST library (b) and (c).

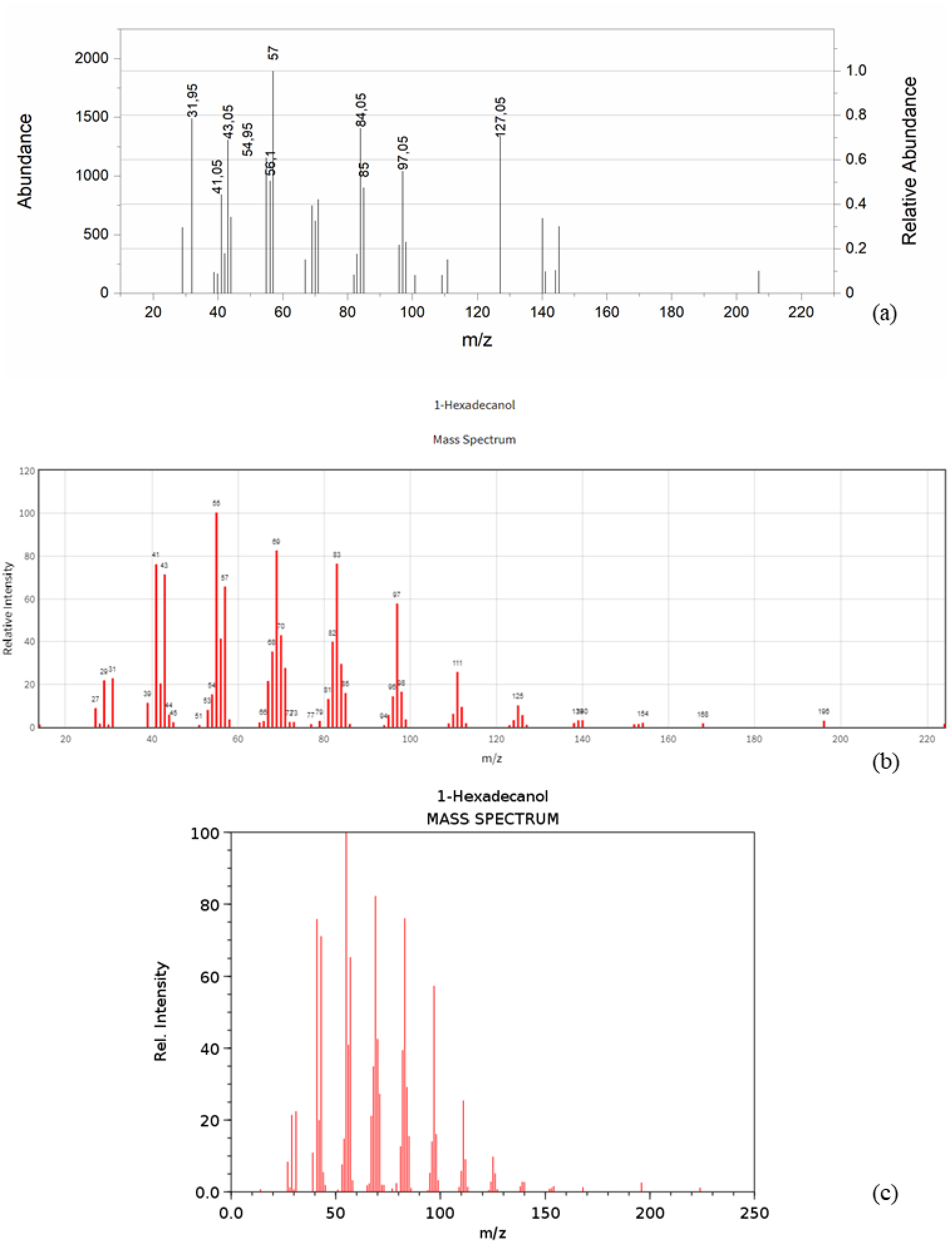

Figure S7 Mass to charge ratio (m/z) plot related to the chromatographic peak with retention time of 58.24 min (a), tentatively identified as hexadecanol, and the m/z plots for the identified compound according to the NIST library (b) and (c).

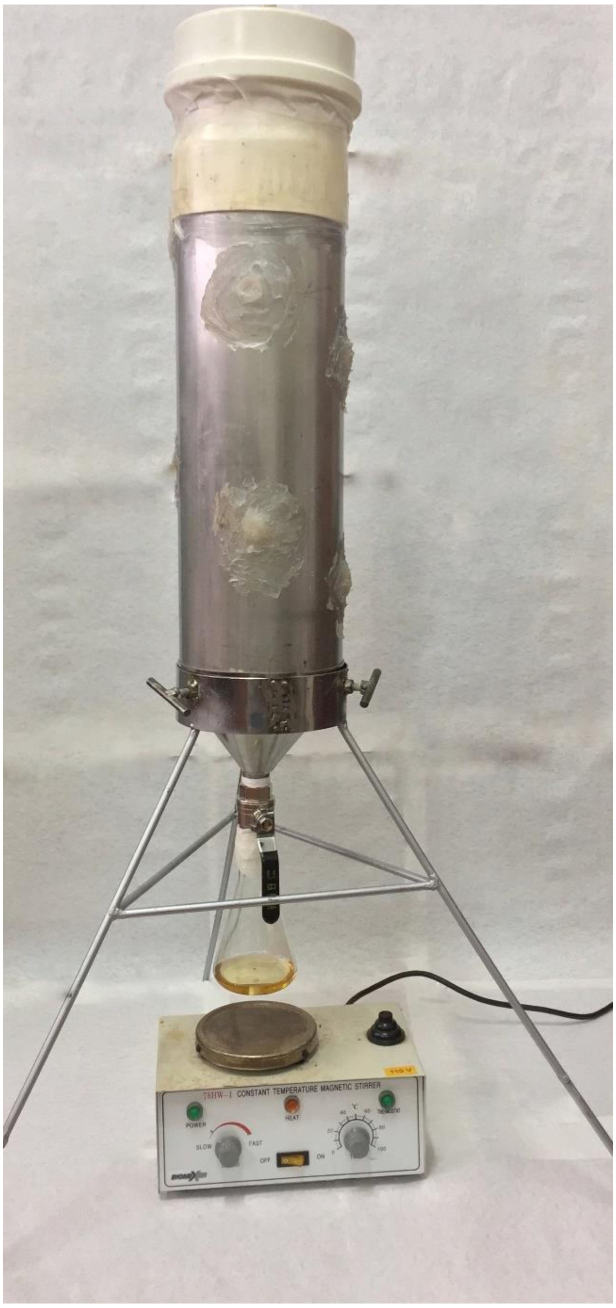

Figure S8 Experimental apparatus used to determine the diffusion coefficient of eugenol through rice.
